# Supplementary material for: How Closely Does Induced Agarwood’s Biological Activity Resemble That of Wild Agarwood?
Source: Molecules. 2023 Mar 24;28(7):2922. doi: 10.3390/molecules28072922 (PMC10096168; doi:10.3390/molecules28072922)
Supplement: Supplementary file 1 [file molecules-28-02922-s001.zip › molecules-2261251-SI.docx]

**Table S1.** Chemical composition of wild agarwood, induced agarwood.

| Induced agarwood (IA) | | | | Wild agrwood (WA) | | | |
| --- | --- | --- | --- | --- | --- | --- | --- |
| RT（min） | Formula | Component | Relative content（%） | RT（min） | Formula | Component | Relative content（%） |
| 6.309 | C_10_H_12_O | 4-phenyl-2-Butanone | 0.31% | 7.873 | C_19_H_28_O_4_ | Pentanedioic acid, (2,4-di-t-butylphenyl) mono-ester | 0.99% |
| 7.98 | C_13_H_16_O_3_ | Ethyl 4-oxo-2-phenylpentanoate | 2.22% | 7.934 | C_17_H_25_NO_2_ | Phenol, 2,6-bis(1,1-dimethylethyl)-4-methyl-, methylcarbamate | 8.11% |
| 9.373 | C_13_H_18_O | alpha-methyl-4-(2-methylpropyl)-Benzeneacetaldehyde | 0.35% | 8.653 | C_12_H_18_O | (4aS,8R)-4a,8-Dimethyl-4,4a,5,6,7,8-hexahydronaphthalen-2(3H)-one | 0.55% |
| 9.518 | C_15_H_24_O | Calarene epoxide | 0.38% | 9.24 | C_17_H_28_O_2_ | 5-Azulenemethanol, 1,2,3,4,5,6,7,8-octahydro-.alpha.,.alpha.,3,8-tetramethyl-, acetate, [3S-(3.alpha.,5.alpha.,8.alpha.)]- | 0.61% |
| 9.612 | C_15_H_24_ | 4-(1,5-dimethyl-1,4-hexadienyl)-1-methyl-Cyclohexene | 0.30% | 9.311 | C_15_H_26_O | 2-((2R,8R,8aS)-8,8a-Dimethyl-1,2,3,4,6,7,8,8a-octahydronaphthalen-2-yl)propan-2-ol | 0.76% |
| 9.754 | C_15_H_22_O | 2-((2R,4aR,8aR)-4a,8-Dimethyl-1,2,3,4,4a,5,6,8a-octahydronaphthalen-2-yl)acrylaldehyde | 0.76% | 9.354 | C_15_H_24_ | Azulene, 1,2,3,5,6,7,8,8a-octahydro-1,4-dimethyl-7-(1-methylethenyl)-, [1S-(1.alpha.,7.alpha.,8a.beta.)]- | 0.48% |
| 9.845 | C_15_H_22_O | 3,4,4a,5,6,7-hexahydro-4a,5-dimethyl-3-(1-methylethenyl)-, [3S-(3.alpha.,4a.alpha.,5.alpha.)]-1(2H)-Naphthalenone | 1.95% | 9.363 | C_15_H_22_O | Isolongifolen-5-one | 0.51% |
| 9.902 | C_15_H_24_O | Humulene epoxide I | 0.55% | 9.558 | C_15_H_26_O | Spiro[4.5]dec-8-en-7-ol, 4,8-dimethyl-1-(1-methylethyl)- | 0.28% |
| 10.124 | C_15_H_24_O | 2-(4a,8-Dimethyl-1,2,3,4,4a,5,6,7-octahydro-naphthalen-2-yl)-prop-2-en-1-ol | 0.50% | 9.724 | C_15_H_24_O | Acorenone B | 2.10% |
| 10.426 | C_15_H_22_O | Ylangenal | 1.18% | 9.82 | C_13_H_18_O | Benzene, 1-cyclohexyl-2-methoxy- | 0.71% |
| 10.46 | C_14_H_18_O_2_ | 1-(2-Methoxyphenyl)-5-methyl-4-hexene-1-one | 0.57% | 10.098 | C_15_H_26_O_2_ | Daucol | 0.60% |
| 10.496 | C_23_H_40_ | 1,3,5-tris(cyclohexyl)pent-1-ene | 0.33% | 10.163 | C_15_H_26_O | Cyclohexanemethanol, 4-ethenyl-.alpha.,.alpha.,4-trimethyl-3-(1-methylethenyl)-, [1R-(1.alpha.,3.alpha.,4.beta.)]- | 0.31% |
| 10.572 | C_29_H_46_ | 24-Noroleana-3,12-diene | 0.43% | 10.356 | C_15_H_22_O | 3-epi-Cedrenal | 0.36% |
| 11.295 | C_13_H_22_O | 1a,2,5,5Tetramethyl-trans-1a,4a,5,6,7,8-hexahydro-gamma-chromene | 0.63% | 10.398 | C_14_H_18_O_2_ | 1-(2-Methoxyphenyl)-5-methyl-4-hexene-1-one | 0.54% |
| 11.908 | C_15_H_26_O_2_ | Arctiol | 0.69% | 10.737 | C_20_H_30_O_4_ | Phthalic acid, butyl 2,4,4-trimethylpentyl ester | 0.39% |
| 12.003 | C_15_H_22_O | 4,5,5a,6,6a,6b-hexahydro-4,4,6b-trimethyl-2-(1-methylethenyl)-2H-Cyclopropa[g]benzofuran | 0.57% | 10.9 | C_15_H_28_O_2_ | 7-(2-Hydroxypropan-2-yl)-1,4a-dimethyldecahydronaphthalen-1-ol | 1.25% |
| 12.392 | C_15_H_22_O_2_ | Valerenic acid | 1.64% | 10.932 | C_12_H_18_O | 2-(5-Isopropyl-2-methylphenyl)ethanol | 0.25% |
| 12.567 | C_15_H_22_O_2_ | 4-(3,3-Dimethyl-but-1-ynyl)-4-hydroxy-3,5,5-trimethyl-cyclohex-2-enone | 0.29% | 11.006 | C_15_H_22_O | 2(3H)-Naphthalenone, 4,4a,5,6,7,8-hexahydro-4a,5-dimethyl-3-(1-methylethylidene)-, (4ar-cis)- | 0.64% |
| 12.829 | C_15_H_22_O_3_ | 5,8-Dihydroxy-4a-methyl-4,4a,4b,5,6,7,8,8a,9,10-decahydro-2(3H)-phenanthrenone | 18.56% | 11.237 | C_15_H_22_O_2_ | (2S,6R)-2,6-Dimethyl-2-(2-(4-methylfuran-3-yl)ethyl)cyclohexanone | 0.21% |
| 12.96 | C_11_H_18_O | 2-Methyl-2-adamantanol | 0.76% | 11.325 | C_15_H_22_O | 5(1H)-Azulenone, 2,4,6,7,8,8a-hexahydro-3,8-dimethyl-4-(1-methylethylidene)-, (8S-cis)- | 9.90% |
| 13.636 | C_14_H_22_O | (+)-Longicamphenylone | 0.69% | 11.405 | C_15_H_24_O_2_ | Spiro[4.5]decan-7-one, 1,8-dimethyl-8,9-epoxy-4-isopropyl- | 0.58% |
| 13.74 | C_15_H_22_O_2_ | 6-(1-Hydroxymethylvinyl)-4,8a-dimethyl-3,5,6,7,8,8a-hexahydro-1H-naphthalen-2-one | 1.64% | 11.478 | C_20_H_30_O_4_ | Phthalic acid, isobutyl 4-octyl ester | 0.34% |
| 13.981 | C_15_H_22_O_2_ | 2aS,3aR,5aS,9bR)-2a,5a,9-Trimethyl-2a,4,5,5a,6,7,8,9b-octahydro-2H-naphtho[1,2-b]oxireno[2,3-c]furan | 0.82% | 11.545 | C_15_H_26_O_2_ | 3.beta.,9.beta.-Dihydroxy-3,5.alpha.,8-trimethyltricyclo[6.3.1.0(1,5)]dodecane | 0.29% |
| 14.064 | C_12_H_12_O_5_ | 2-(2-Hydroxypropionyl)phenylglyoxylic acid, methyl ester | 1.2% | 11.706 | C_20_H_40_O_3_ | Carbonic acid, decyl nonyl ester | 0.23% |
| 15.208 | C_17_H_16_O | 1,5-diphenyl-1-Penten-3-one | 0.35% | 11.832 | C_14_H_20_O_3_ | 1-(5-Hexyl-2,4-dihydroxyphenyl)ethanone | 0.28% |
| 15.657 | C_15_H_18_O_2_ | 2-hydroxy-5-(3-methyl-2-butenyl)-4-(1-methylethenyl)-2,4,6-Cycloheptatrien-1-one | 0.29% | 11.869 | C_15_H_26_O_2_ | 1,1,4,7-Tetramethyldecahydro-1H-cyclopropa[e]azulene-4,7-diol | 4.27% |
| 17.216 | C_17_H_14_O_2_ | 2-(2-phenylethyl)chromone | 9.46% | 12.129 | C_17_H_24_O_3_ | 7,9-Di-tert-butyl-1-oxaspiro(4,5)deca-6,9-diene-2,8-dione | 0.21% |
| 19.43 | C_17_H_14_O_4_ | 7-Methoxy-3-(p-methoxyphenyl)chromone | 3.06% | 12.374 | C_14_H_24_O | 2,4a,5,8a-Tetramethyl-1,2,3,4,4a,7,8,8a-octahydronaphthalen-1-ol | 8.76% |
| 20.09 | C_18_H_16_O_3_ | 8-Methoxy-2-(2-phenylethyl)chromone | 8.39% | 12.502 | C_16_H_32_O_2_ | n-Hexadecanoic acid | 0.46% |
| 20.156 | C_18_H_17_ClO_5_ | Succinic acid, 3-chlorophenyl 4-methoxybenzyl ester | 5.55% | 12.525 | C_12_H_18_O | 2-(1-Cyclohexenyl)cyclohexanone | 1.37% |
| 20.653 | C_23_H_20_ | 1-[4,4-diphenyl-1,3-butadien-1-yl]-4-methyl-benzene | 2.02% | 12.593 | C_16_H_22_O_4_ | Dibutyl phthalate | 0.40% |
| 21.781 | C_15_H_14_O_3_ | 1-Acetyl-2-hydroxy-4-benzyloxybenzene | 1.37% | 12.656 | C_16_H_26_O_2_ | 3-Heptyn-2-one, 5-cyclopentyl-6-hydroxy-6-methyl-5-(1-methylethyl)- | 2.46% |
| 22.261 | C_30_H_50_ | Supraene | 0.35% | 12.755 | C_15_H_22_O_3_ | 5,8-Dihydroxy-4a-methyl-4,4a,4b,5,6,7,8,8a,9,10-decahydro-2(3H)-phenanthrenone | 2.73% |
| 22.469 | C_19_H_18_O_4_ | 6-Methoxy-2-(4-methoxyphenethyl)chromone | 2.15% | 12.892 | C_16_H_34_O | 3-Isopropyl-6,10-dimethylundecane-2-ol | 0.21% |
| 22.886 | C_19_H_18_O_4_ | 6,7-Dimethoxy-2-(2-phenethyl)chromone | 23.17% | 12.962 | C_15_H_22_O_3_ | 5,8-Dihydroxy-4a-methyl-4,4a,4b,5,6,7,8,8a,9,10-decahydro-2(3H)-phenanthrenone | 3.36% |
| 25.286 | C_20_H_20_O_5_ | 6,7-Dimethoxy-2-(4-methoxyphenethyl)chromone | 2.53% | 13.079 | C_15_H_24_O_2_ | (4aS,7R)-7-(2-Hydroxypropan-2-yl)-1,4a-dimethyl-4,4a,5,6,7,8-hexahydronaphthalen-2(3H)-one | 2.52% |
|  |  |  |  | 13.59 | C_15_H_22_O_2_ | 6-(1-Hydroxymethylvinyl)-4,8a-dimethyl-3,5,6,7,8,8a-hexahydro-1H-naphthalen-2-one | 1.40% |
|  |  |  |  | 13.705 | C_15_H_22_O_2_ | Valerenic acid | 1.64% |
|  |  |  |  | 13.86 | C_12_H_16_O_2_ | Pentamethylbenzoic acid | 0.35% |
|  |  |  |  | 13.952 | C_15_H_22_O_2_ | 2aS,3aR,5aS,9bR-2a,5a,9-Trimethyl-2a,4,5,5a,6,7,8,9b-octahydro-2H-naphtho[1,2-b]oxireno[2,3-c]furan | 2.09% |
|  |  |  |  | 14.085 | C_15_H_18_O_2_ | 2,4,6-Cycloheptatrien-1-one, 2-hydroxy-5-(3-methyl-2-butenyl)-4-(1-methylethenyl)- | 1.32% |
|  |  |  |  | 14.095 | C_17_H_32_O_3_ | Carbonic acid, tetradecyl vinyl ester | 0.24% |
|  |  |  |  | 15.016 | C_11_H_12_O_3_ | Ethyl 4-acetylbenzoate | 0.33% |
|  |  |  |  | 15.44 | C_23_H_46_O_2_ | Heneicosyl acetate | 0.27% |
|  |  |  |  | 17.695 | C_22_H_42_O_4_ | Hexanedioic acid, bis(2-ethylhexyl) ester | 19.46% |
|  |  |  |  | 17.993 | C_23_H_32_O_2_ | Phenol, 2,2'-methylenebis[6-(1,1-dimethylethyl)-4-methyl- | 4.54% |
|  |  |  |  | 18.666 | C_16_H_34_O | 1-Hexadecanol | 0.30% |
|  |  |  |  | 19.341 | C_24_H_38_O_4_ | Phthalic acid, di(2-propylpentyl) ester | 0.31% |
|  |  |  |  | 22.197 | C_30_H_50_ | Supraene | 0.27% |
|  |  |  |  | 22.84 | C_19_H_18_O_4_ | 6,7-Dimethoxy-2-phenethyl-4H-chromen-4-one | 1.16% |

**Figure S1.** Standard curve for the determination of total chromones content.

| 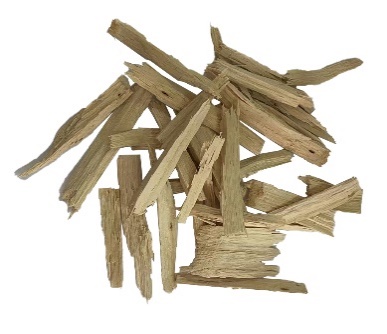 | 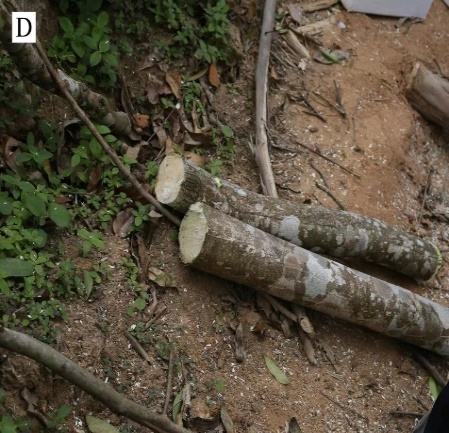 |
| --- | --- |

**Figure S2.** Healthy uninoculated *A. sinensis.*
